# The role of social vulnerability in improving interventions for neglected zoonotic diseases: The example of Kyasanur Forest Disease in India

**DOI:** 10.1371/journal.pgph.0000758

**Published:** 2023-02-08

**Authors:** Festus A. Asaaga, Bethan V. Purse, Mujeeb Rahman, Prashanth N. Srinivas, Suresh D. Kalegowda, Tanya Seshadri, Juliette C. Young, Meera A. Oommen

**Affiliations:** 1 UK Centre for Ecology & Hydrology, Wallingford, United Kingdom; 2 Ashoka Trust for Research in Ecology and the Environment, Bengaluru, Karnataka, India; 3 Institute of Public Health, Bengaluru, Karnataka, India; 4 National Institute of Veterinary Epidemiology and Disease Informatics, Bengaluru, Karnataka, India; 5 Agroécologie, INRAE, Institut Agro, Univ. Bourgogne, Univ. Bourgogne Franche-Comté, Dijon, France; 6 Tribal Health Resource Center, Vivekananda Girijana Kalyana Kendra, BR Hills, Karnataka, India; COMSATS University Islamabad, PAKISTAN

## Abstract

Forest-based communities manage many risks to health and socio-economic welfare including the increasing threat of emerging zoonoses that are expected to disproportionately affect poor and marginalised groups, and further impair their precarious livelihoods, particularly in Low-and-Middle Income (LMIC) settings. Yet, there is a relative dearth of empirical research on the vulnerability and adaptation pathways of poor and marginalised groups facing emerging zoonoses. Drawing on a survey of 229 households and a series of key-informant interviews in the Western Ghats, we examine the factors affecting vulnerability of smallholder and tribal households to Kyasanur Forest Disease (KFD), an often-fatal tick-borne viral haemorrhagic fever endemic in south India. Specifically, we investigate how different socio-demographic and institutional factors interact to shape KFD vulnerability and the strategies employed by households to adapt to disease consequences. Although surveyed households generally perceived KFD as an important health issue in the study region, there was variability in concern about contracting the disease. Overall results showed that poor access to land (AOR = 0.373, 95% CI: 0.152–0.916), being at or below the poverty line (AOR = 0.253, 95% CI: 0.094–0.685) and being headed by an older person (AOR = 1.038, 95% CI: 1.006–1.071) were all significant determinants of perceived KFD vulnerability. Furthermore, KFD vulnerability is also modulated by important extra-household factors including proximity to private hospitals (AOR = 3.281, 95% CI: 1.220–8.820), main roads (AOR = 2.144, 95% CI: 1.215–3.783) and study location (AOR = 0.226, 95% CI: 0.690–0.743). Our findings highlight how homogenous characterisation of smallholder and tribal communities and the ‘techno-oriented’ approach of existing interventions may further marginalise the most vulnerable and exacerbate existing inequalities. These findings are important for designing context-specific and appropriate health interventions (including the prioritisation of awareness raising, knowledge networks, livelihood diversification) that enhances the resilience of at-risk social groups within the KFD context. More broadly, our findings highlight how a focus on social vulnerability can help national and international health planners improve health interventions and prioritise among diseases with respect to neglected endemic zoonoses.

## 1. Introduction

Kyasanur Forest Disease (KFD) is a tick-borne viral zoonotic disease transmitted to humans through infected tick bites (primarily Haemaphysalis spinigera) within agro-forestry mosaic in south India. Whereas KFD has a relatively low mortality rate (3–10% mortality) with yearly incidence of about 500 cases per annum [1–3], it is a recognised disease of public health concern in India, with substantial and disproportionate livelihood impacts on small-holder and tribal forest-dependent communities [4–6]. While statistics on the disease burden is patchy, it is estimated that about 9,594 cases of KFD occurred in 16 districts from 1957 to 2017 [7]. At present there is no cure for KFD, and the efficacy of the vaccine in mitigating outbreaks is contested [8, 9]. Although research on the diagnostics and treatment protocols is paramount, reduction of the disease burden is also contingent on reducing the number of new infections. Since its discovery in March 1957 in the Kyasanur forest in Soraba *taluk* (a sub-district in India) of Shivamogga district in Karnataka state [10], the geographical distribution has expanded considerably to adjacent states along the Western Ghats namely, Kerala, Goa, Maharashtra and Tamil Nadu [7, 11–13]. The growing recognition of the widespread regional susceptibility to KFD and its associated risk factors (e.g. limited disease awareness, degradation and human use of forests, poor diagnostics and surveillance) has amplified interest in developing resilience in health systems (in regards improved disease surveillance and control measures) in India [14].

Considerable research on KFD in the last century focussed on the clinical symptoms and treatments [8] and the transmission ecology in the period following initial emergence. However, there remain important knowledge gaps on ecological drivers of the disease transmission and spread [2, 15], and the bio-cultural, political and ecological dynamics which underpin the capacity of populations affected to cope with and adapt to KFD and its associated stressors [4, 7, 15, 16]. This appears to echo an important lacuna in wider public health literature, namely understanding the role of social structures and human agency in (re-)producing vulnerability and coping or adapting to zoonotic diseases [16–18].

Against this background, this paper seeks to add to the existing body of knowledge by examining households’ vulnerability to KFD using a political ecology approach. The analysis draws on a multi-dimensional conceptualisation of vulnerability that includes exposure, sensitivity and adaptive capacity to examine how these different elements of vulnerability interact to shape or modulate the overall susceptibility of small-holder and tribal forest-dependent households’ exposure to KFD.

The paper is organised as follows. Section 2 presents a synthesis of the literature on conceptualising vulnerability using a political ecology approach, which provides a theoretical foundation to situate the subsequent empirical analysis. This is followed by a critical contextual overview of KFD management in India, outlining the case for addressing vulnerability to the disease in Section 3. In particular, we identify how a focus on vulnerability can contribute to the development of targeted OneHealth interventions that foster local adaptive capacity. Section 4 describes the methods after which findings on the main determinants of KFD vulnerability in the study context are presented in Section 5. The paper concludes by reflecting on the implications of the study.

## 2. Conceptualising the political ecology and vulnerability nexus—An overview

**Fig 1:**
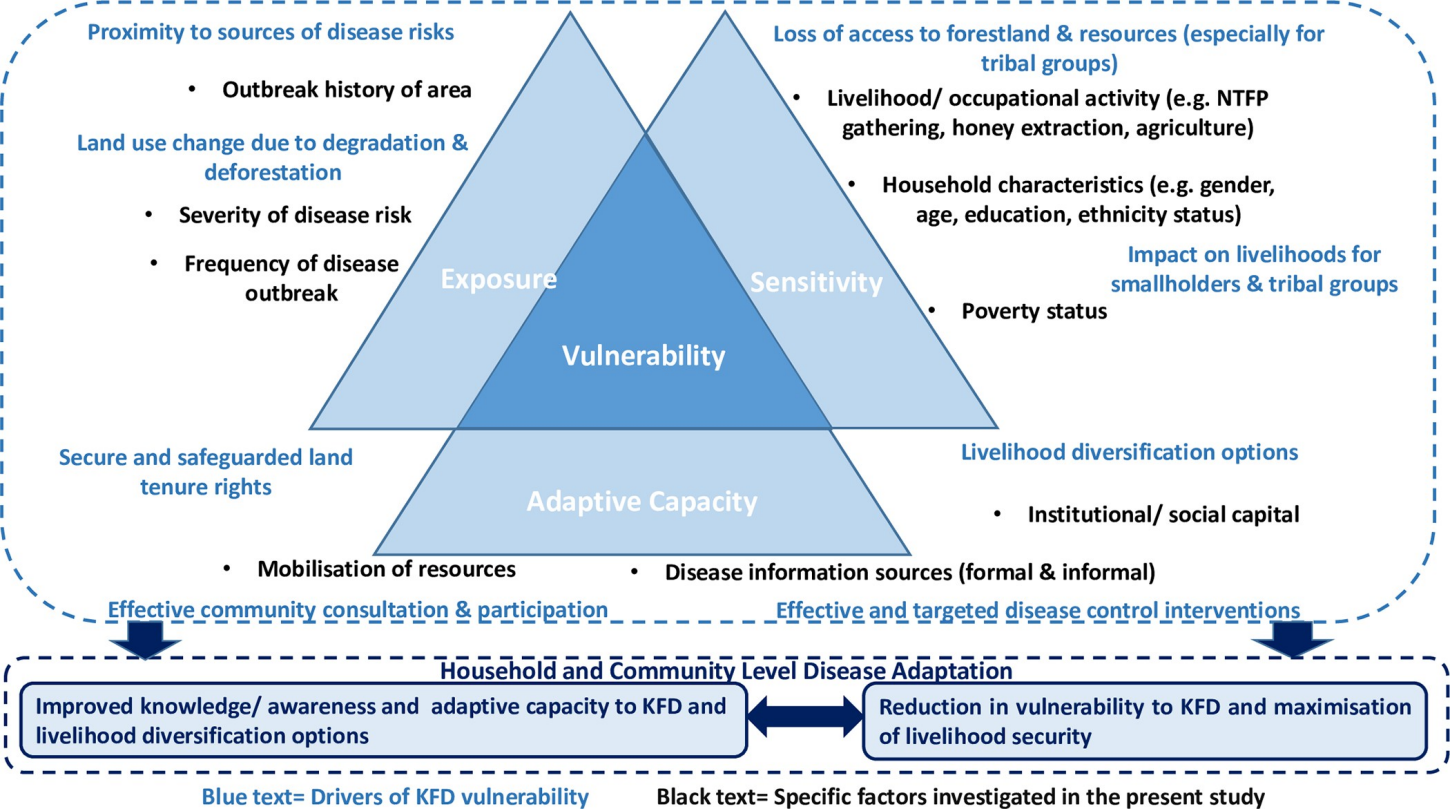
Multi-dimensional zoonotic disease vulnerability in a socio-ecological setting based on modification of Mitchell & McEvoy [27].

There is an increased impetus for understanding the contextual dynamics of vulnerability to emerging disease risks in the health policy and planning scholarship. Despite the growing consensus that smallholders are disproportionately impacted, the context of their individual and/ or household vulnerabilities occasioned by exposure to disease hazards are still poorly understood, particularly in developing contexts [4, 17]. Different empirical observations have reported that household vulnerabilities are often predicated on myriad of social, cultural, economic, institutional and biophysical factors that surround disease dynamics, necessitating holistic frameworks to better understand differential patterns of exposure to disease hazards [19–22]. The political ecology approach is highly advocated as a plausible framework to unpack the interplay of contextual and institutional factors influencing individual or household vulnerabilities and their relative capacity to implement changes in response to disease stressors [20, 21, 23]. Hakansson [20] for instance, observes that the political ecology approach elucidates the complexities of human-environmental interrelationships as a central aspect of differential distribution of power, economic inequality, property relations, and entitlements overtime [21, 23]. In particular, disease vulnerability can be understood as a heterogeneous and temporal characteristic of differentiated cultural or social units, mediated by several interacting factors [20, 21, 24], [Fig 1]. A political ecology perspective therefore offers an analytical lens to critically elucidate the human-environmental interrelationships ‘through a focus on hierarchically organised resource management, in which social groups and individuals are differentially situated in institutions and networks that extend well outside of the local arena’, within the human-animal-environment interface [20, 25]. In this sense, households’ experiences with KFD and its associated stressors or vulnerabilities can be understood as arising within the domain of a structured and hierarchical institutional context, filtered through the broader political economy of power, social and physical capital and access to and use of the communal forest resources [25, 26].

Investigating the dynamics of household vulnerability to KFD in the Western Ghats region, the present study adopts a multidimensional conceptualisation of vulnerability. Previously, the concept of vulnerability has been variously defined with no consensus on what it constitutes, let alone the means of measuring it [28, 29]. The term ‘vulnerability’ often tends to connote different things to different scholar-practitioner communities depending on their theoretical or disciplinary orientation [28–31]. Of the several definitions proposed, the crosscutting view is that vulnerability is a dynamic phenomenon that is experienced contextually [28, 30], and that its measurement should necessarily reflect the circumstances ‘on the ground’ [28].

As a point of departure, this study defines vulnerability as the susceptibility of an individual or household to respond negatively to a disease stress and/or associated impacts generally due to the lack of capacity to cope. In this sense, social vulnerability in the context of disease management is understood as the degree to which groups of people or individuals perceive their susceptibility to the actual or potential impacts of a disease stress and their capacity to cope with adverse effects on their livelihoods and welfare, following Adger [28]. Stressors, in this sense, encompasses disruption to groups or individuals’ livelihoods and forced adaptation to the changing conditions occasioned by exposure to a disease risk [28]. As illustrated in Fig 1, social vulnerability is understood as a function of exposure to a disease hazard, in this case KFD, and the sensitivity of the exposed individual or household in relation to the hazard (e.g. occupational activity, poverty status etc.) counteracted by adaptive capacity (e.g. information access, secure tenure rights etc.) [Fig 1]. Building on Mitchel & McEvoy [27] tripartite conceptualisation of vulnerability, exposure in this context, for instance, refers to the nature of disease risk which affects directly or indirectly health and livelihood outcomes. The sensitivity dimension relates to the organisation and structure of the local system relative to disease-related outcomes and determines the severity at which exposure manifest [22]. For instance, differences in household occupational activities and poverty status will determine the degree of impact when two households are exposed in the event of a disease outbreak [27], [Fig 1]. Adaptive capacity in this context is the ability to address, plan for, or adapt to adverse disease-related outcomes and take advantage of new opportunities and benefits [28, 32]. Fig 1 typifies factors such as access to disease information, enabling governance structures and access to healthcare determine the potential of an individual or household to adapt in the face of emerging disease risks.

Prior work [30, 32, 33] distinguishes between vulnerability of outcome and contextual vulnerability. O’Brien et al. [30] for instance, argue that a contextual (‘place-based’) framing of vulnerability allows a holistic analysis of socio-ecological systems, particularly how socially and biophysically mediated factors can interact to reduce adaptive capacity, as opposed to focussing on how impacts of an external perturbation (in this case KFD) manifest and, thus, inadvertently create a focus on technical interventions [32]. Community-focused scholars [28, 34] contend that the underlying social structures are fundamental to understanding contextual vulnerability and household and community level adaptation pathways. As Sewell [35] explains, social structures are ‘sets of mutually sustaining schemas (patterns of thoughts and behaviours) and resources that tend to be reproduced by that social action’. These social structures include, class, ethnicity, education, norms and customs, as well as forms of political and economic organisation [32, 33]. Thus, focussing on a contextual vulnerability from a tripartite perspective [Fig 1] affords a nuanced understanding of vulnerability by moving beyond developing purely structural explanations to interrogating the ways in which ecological and bio-physical factors shape, and are shaped by, the variety of relevant socio-cultural, political and economic structures and ultimate household and/or community level knowledge, health and adaptation outcomes [33], [Fig 1].

## 3. Problematizing KFD management in India–a critical overview

An understanding of vulnerability to KFD, characterised as a ‘neglected disease of poverty’ [36], requires familiarity with the broader institutional context of KFD control and zoonoses management in India. Within the zoonoses literature, there is strikingly very limited empirical engagement on the differential patterns of vulnerability with respect to neglected zoonoses and the links to adaptive actions. In the resulting reductionist perspectives lead to limited impact of disease control interventions due to issues such as paucity of data on disease burdens and impacts on different social groups [17, 19].

Against this background, the context of KFD management in India presents an interesting case for understanding how the interplay of bio-cultural and institutional factors operate to shape patterns of vulnerability and outcomes of disease control interventions at the local level. Despite India’s history of response to important zoonotic disease risks [37], the country struggles with the threat of endemic zoonotic pathogens, with considerable disproportionate impacts on marginalised forest communities [4, 11, 15]. As a known hotspot for zoonotic diseases, India’s disease system has over time witnessed the implementation of several disease control programmes across the public health and animal health sectors at the national (e.g. National Standing Committee on Zoonoses (NSCZ)) and sub-national levels (Integrated Disease Surveillance Programme (IDSP) and National Vector-borne Diseases Control Programme (NVBDCP)) geared towards combating the threat from both exotic and endemic zoonotic pathogens. However, several scholars have criticised interventions especially around neglected zoonotic diseases as often *ad hoc* and reactive [37, 38]. Among several criticisms, the limited cross-sectoral engagement between the human and animal health sectors on one hand, and the environment sector on the other, fragility of local health systems, and low disease prioritisation and funding challenges have been observed to significantly hamper the success of interventions [4]. For KFD there is widespread consensus that cross-sectoral understanding across the human-animal-environment interface is critical to effective control [39]. However, as is commonly found for zoonotic diseases [40, 41] recent studies have highlighted that existing interventions largely focus on humans (e.g. vaccination, treatment) overlooking animal hosts, or the environmental settings within which spill-over to humans occurs [42]. While these limitations may echo the frailties of the wider health system, they are particularly pronounced in the case of neglected endemic zoonoses of poverty such as KFD and scrub typhus, with a disproportionately high impact on forest-dependent communities and low prioritisation at the national level [4]. The recent considerable shifts in the KFD geographical range vis-à-vis the lack of specific treatment [2, 39, 43] has led to its reprioritisation from a localised problem (affecting few states) to a national public health concern [4, 39].

At the same time, there is a growing consensus within the theoretical and policy circles on the importance of an institutionalised ‘One Health’ approach to tackle the continuing threat of KFD and other neglected zoonotic pathogens in India [38, 44, 45]. Central to this perspective is the recognition of the complex disease ecology with disproportionate impact on different marginalised social groups, shaped by caste, gender, socio-economic status and other exogenous factors (e.g. healthcare access) operating to create differential exposure and produce specific types of vulnerabilities [19, 22]. As Bardosh et al. [19] observed, different social groups react differently to disease control strategies given the differential capacities that may exist within and across groups. This is especially so in the case of smallholder and tribal groups who are particularly sensitive to zoonotic disease risks due to their resource-based livelihoods and traditionally-oriented health systems [22].

Previous research has highlighted that smallholder farmers, plantation and forestry workers and tribal groups dependent on forest resources for their livelihoods are particularly vulnerable with a substantial risk of exposure to tick-borne infections (including KFD), which underscores the rationale of identifying and minimising disease risks within and across such groups [6–9, 13]. To date, there is no evidence of human-to-human transmission of KFD [13], and humans are “dead-end” hosts. Thus vaccination remains an important aspect of the existing control strategy for the disease for reducing clinical impacts in infected individuals, given the potentially high-risk of exposure associated with forest-based livelihood activities [4, 13]. Yet the effectiveness of the policy of using an old formalin inactivated tissue-culture vaccine, that requires multiple doses, in reducing disease impacts is widely contested [8, 9, 11]. Kasabi et al. [8] reported low vaccination coverage in five districts of Karnataka (between 2005 and 2010) with just 36% of the target population receiving two doses and a booster dose, which conferred about 83% immunity. Oliveira et al. [11] reported that individuals who received just one or two doses were still as susceptible to contracting KFD as those unvaccinated, though clinical impacts were reduced. Other studies have highlighted the importance of understanding the nexus between knowledge sharing and adaptation measures by different social groups (particularly by mobile and migrant populations) in modulating vulnerability to zoonotic diseases [4].

Responding to the social difference in KFD transmission and control remains therefore a central component of alleviating and/ or mitigating future disease situations [17, 19]. This requires a better understanding of the patterns of vulnerability and factors that shape key outcomes. While considerable success has been achieved in fostering cross-departmental collaboration during KFD outbreak situations, these actions are still sporadic and have yet to result in any consensus on the form of cross-sectoral coordination [39]. Indeed, on-going discussions on KFD interventions have tended to overly emphasise the importance of technical and bio-medical expertise (around vaccine development and treatment) to the relative neglect of equally critical aspects such as the capacity of vulnerable populations to cope/ adapt to the emerging disease risks, which remain at the periphery of present policy deliberations. Although KFD has immediate and proximal biological causes, the vulnerability to the disease, and indeed the mechanisms leading to exposure are often not biological, but originate in the social circumstances in which people live and work and the relationships between groups and individuals [4, 46]. To guide on-going and future disease interventions, this paper addresses the need to move beyond static descriptions to investigate the dynamic and relational understandings of the changing disease ecology and the social configurations that influence vulnerability as well as the differential disease vulnerability that exists within and across key social groups.

## 4. Materials and methods

### 4.1 Study areas

**Fig 2:**
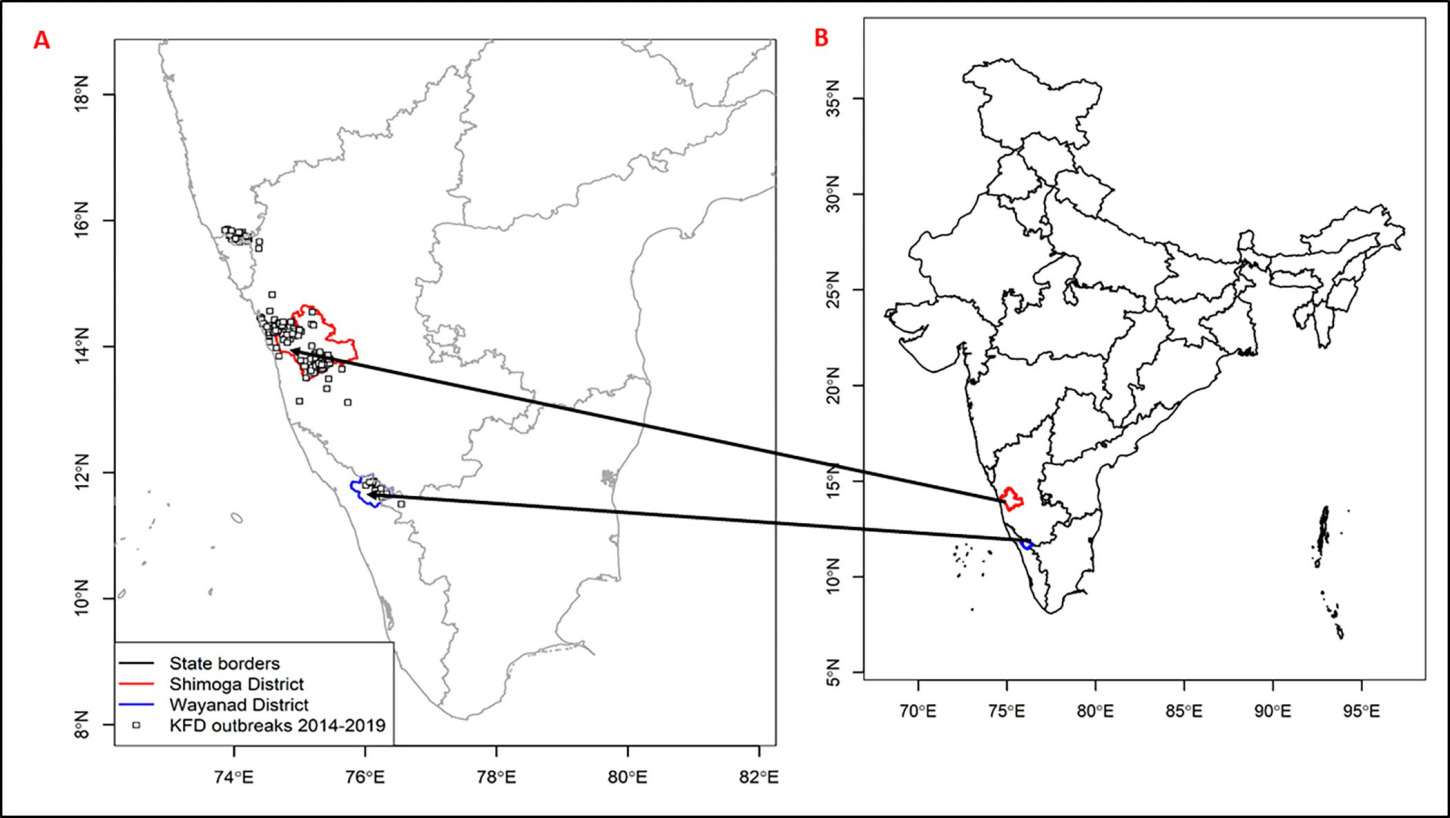
Map of the Western Ghats depicting the locations of Shivamogga (a KFD endemic district) and Wayanad (emergent district) within India, and reported KFD outbreak cases (2014–2019). Adapted from Asaaga et al. [4] and Purse et al. [15]. Source data: Map base layer is from the OpenStreetMap (https://wiki.openstreetmap.org/wiki/Standard_tile_layer). This dataset is available under a CC0 1.0 Universal (CC0 1.0) Public Domain Dedication license (https://creativecommons.org/publicdomain/zero/1.0/) and any copy of or work based on this dataset requires the following attribution: This dataset is based on the dataset produced by the OpenStreetMap Foundation (https://osmfoundation.org/). The administrative boundary dataset used in this figure is from HindudstanTimesLabs (https://github.com/HindustanTimesLabs/shapefiles/), reproduced under the MIT License. Human case data are from the Department of Health and Family Welfare Services, Government of Karnataka.

Shivamogga and Wayanad districts are both situated in the Western Ghats region, which covers an area of 140,000 km^2^, stretching 1,600 km from the south of the Tapti river (near the border of Gujarat and Maharashtra) traversing the states of Kerala, Tamil Nadu, Karnataka, Goa, Maharashtra and Gujarat [15] [see Fig 2]. The Western Ghats is one of the world’s eight ‘hottest biodiversity hotspots’, with several wildlife sanctuaries and dense moist evergreen forest reserves, which surrounding small-holder and tribal communities depend on for their livelihoods. Given its immense socio-ecological significance, the Western Ghats was listed as UNESCO protected World Heritage Site in 2012. The region’s complex geography, wide variation in rainfall (1000 to 6000 mm per annum) and altitudinal decrease in temperature, coupled with anthropogenic factors have produced a variety of vegetation types, ranging from evergreen and semi-evergreen forests, to moist-deciduous and scrub [15, 47]. The forests of the Western Ghats have declined considerably over the last two centuries, due to timber extraction, industrial development (roads, railways, dams and mines) and increases in agriculture and plantations [15, 47, 48]. Although data on land use change in the region is patchy, recent forest cover analysis suggests a decline in the area of forest cover, from 73.1% in 1920 to 47.1% in 2013 [48]. Though the decline in forest cover is not ubiquitous [49], focal districts in the Western Ghats districts (e.g. Shivamogga, Wayanad) have experienced reduction in forest cover from 1991 to 2018 [48]. In Shivamogga for instance, Ramachandra et al. [50] estimated a decline in forest coverage from 43.83% of the district (in 1973) to 34.02% in 2018 [15]. Forest loss and habitat fragmentation have been linked with increased KFD emergence and transmission [2, 51], suggesting that changes in land use may increase spatial overlap between human activity (particularly forest visits for farming, hunting, livestock grazing and NTFP collection), wildlife hosts, and ticks [6–8, 12]. Both Shivamogga and Wayanad have high concentration of Adivasis and other tribal communities (e.g. Kurichias, Paniyas, Kattunuayakans), the majority of whose socio-cultural organisation and livelihood security are inextricably linked to ‘secure’ access to and use of forest resources [25, 52–54]. Yet, the abrupt shifts in land use between forest and village settlements (produced by degradation and fragmentation of the forest landscape) potentially increases these communities’ vulnerability to KFD given the close proximity to vectors and wildlife hosts implicated in the disease transmission [15].

### 4.2 Ethical approval and consent to participate

The study was approved by the Institutional Review Boards of the Ashoka Trust for Research in Ecology and the Environment (IRB/CBC/0003/ATV/07/2018) and the Institute of Public Health Bangalore (IEC-FR/04/2017) in India, as well as the Liverpool School of Tropical Medicine (LSTM) Research Ethics Committee (17–062) in the United Kingdom under the MonkeyFeverRisk project [55]. All study participants were adult (≥18 years). Informed (verbal) consent was obtained from all participants prior to the start of the interviews and administration of the survey questionnaire. The rationale for obtaining verbal consent was due to the limited capacity of the study participants to read and write in neither the local language nor English. The collated data were anonymised using de-identifiers or pseudonyms (e.g. SA1, WD1) to protect the privacy of study participants.

#### 4.2.1 Inclusivity in global research

Additional information regarding the ethical, cultural, and scientific considerations specific to inclusivity in global research is included in S1 Checklist.

### 4.3 Data collection

**Fig 3:**
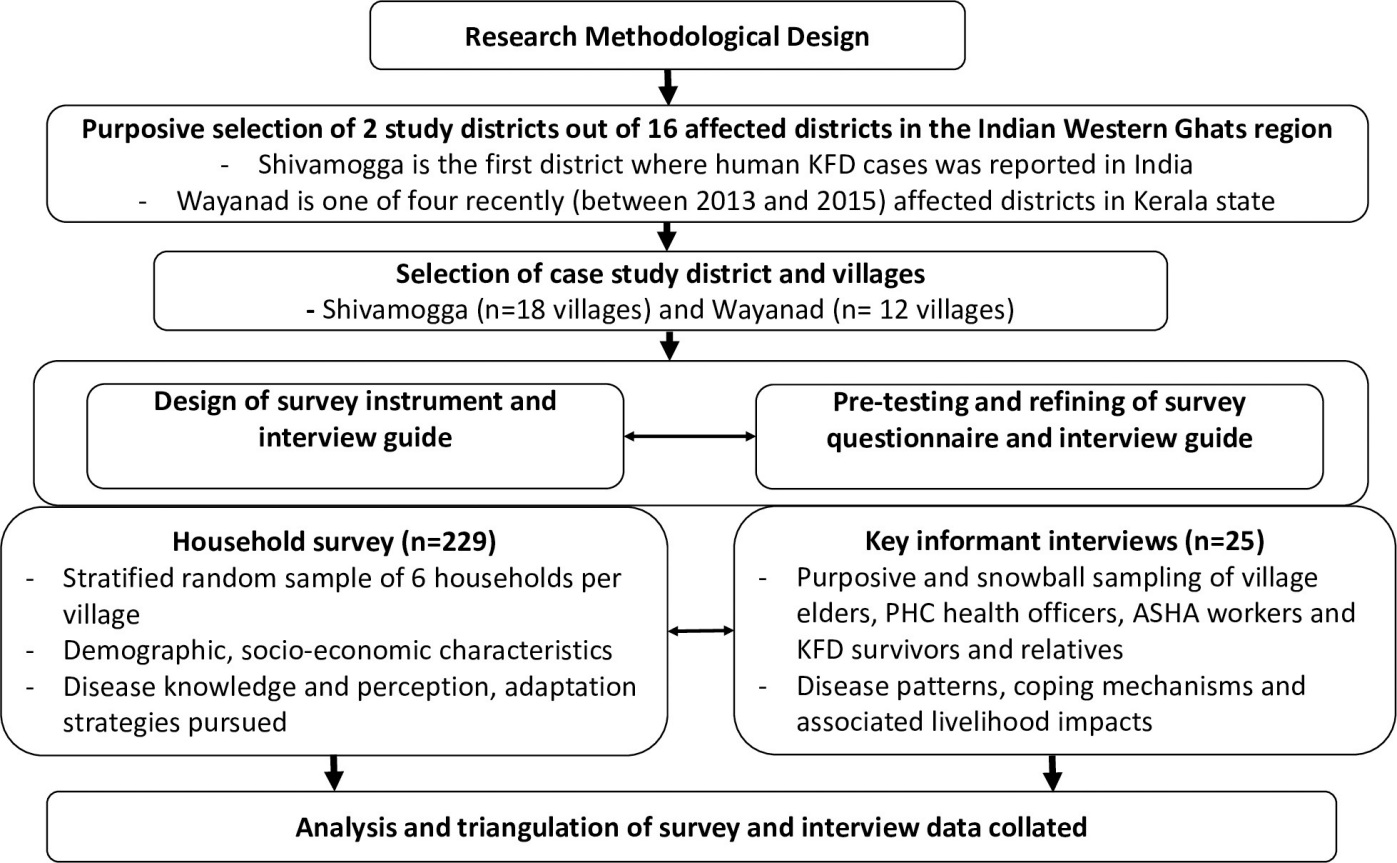
Schematic representation of the research strategy.

Data underpinning the study were collected using a combination of household survey and key informant interviews within a convergent parallel mixed methods design framework. Fig 3 illustrates the research methods and data collection approach that contribute to the dataset under investigation here.

#### 4.3.1 Household survey

A structured questionnaire was used to solicit information from smallholder and tribal forest households to assess their livelihood options and extent of vulnerability to KFD and its associated stressors at the household and community scales. We adopted a stratified multi-stage sampling approach, consisting of four inter-linked stages in the selection of our focal districts, villages and households. First, we purposively selected the two focal states, Karnataka and Kerala to represent the KFD-affected regions along the Western Ghats. This was followed by the selection of specific districts (i.e. Shivamogga and Wayanad) based on their relative disease outbreak histories, with Shivamogga being an endemic district (long-affected) and Wayanad a recently affected district respectively. Aside from their outbreak histories, the relative socio-cultural, economic and political differences of the two study districts afforded the opportunity to explore other important place-based differences that influence patterns of vulnerability within and across social groups. Study villages were selected based on previous disease outbreak history (i.e. present and absent) and proximity (i.e. close and far) to the nearest forest fragments. Present and absent classes were specified as villages that had either experienced or not experienced a KFD outbreak within the last year (2018/19 season) respectively, whereas close and far distance classes were defined as < 1 km and >3 km to large forest fragments (at least 500m^2^) respectively. All the villages within the target districts (Shivamogga and Wayanad) were grouped into two categories–i.e. affected and unaffected, based on which 30 villages were randomly selected to be equally balanced across the disease categories and forest proximity categories. Households within the sampled villages were subsequently selected by a stratified random sampling approach, targeting male-headed and female-headed households that had access to forest. Overall, 229 household surveys were conducted in 30 villages in Shivamogga (n = 18) and Wayanad (n = 12) respectively. The reliability and content validity (i.e. survey contains questions which cover all aspects of the construct being measured) of the final version of the structured questionnaire was assessed by experts in the fields of public health, tribal health, disease ecology, participatory epidemiology and forest governance (from the MonkeyFeverRisk project team). A team of four research assistants were trained who in turn piloted the survey instrument with 10 heads of households to check the acceptability, clarity and relevance of the questions. The research team based on feedback from the pilot study further refined the survey instrument (the pilot household data is excluded from this analysis). The research assistants administered the survey instrument in the local languages, predominately *Kannada* and *Malayalam*, the predominant local languages spoken in Shivamogga and Wayanad respectively. The household surveys addressed knowledge and perceptions about KFD, different patterns of vulnerability, and impact on livelihood dynamics in order to understand the lived experiences and realities of households in the KFD-prone areas. Specifically, we collated data on: household socio-demographic characteristics (age, ethnicity, education, and gender), land tenure arrangements, forest access and use, household assets and income, disease perception and knowledge, adaptation and different adaptive strategies pursued. For further details, see Asaaga et al. [4].

#### 4.3.2 Key informant interviews

To better understand disease patterns, lived experiences, coping mechanisms and associated livelihood impacts, KFD survivors in the focal communities were contacted for key informant interviews. This was premised on the understanding that this cohort had a first-hand experience with KFD and were better positioned to provide relevant information. Village elders and tribal chiefs, district and taluka disease managers and other healthcare workers, and individuals with familial or social connection to persons with KFD were also interviewed. Overall, twenty-five (25) key informant interviews (KII) were conducted in Shivamogga (n = 17) and Wayanad (n = 8) between August 2019 and March 2020. The key informant interviews afforded the opportunity for an in-depth discussion and corroboration of the main issues that were highlighted in the household surveys (see supplementary data file). The key informant interviews were conducted in *Kannada* and *Malayalam* on a one-to-one basis at a location of respondents’ preference and lasted between 45 minutes and one hour [4].

### 4.4 Data analysis

#### 4.4.1 Statistical analysis of households’ vulnerability

Survey data were recorded as hard copies and then entered into a Microsoft Excel 2013 spreadsheet. The dataset was coded, checked for integrity and exported to SPSS (version 20) for analysis. To allow for descriptive comparisons, mean scores and modes were computed for a selection of perception and knowledge variables for the household survey. Pearson chi-squared test was used to assess significant differences (p<0.05) between groups based on the study area and their demographic and socio-economic characteristics. An adaptation score (null, low, medium, high) was developed based on five items assessed (for a maximum of 5 positive responses as a function of participants’ adopted preventive measures to KFD): (null (0 = no positive or ‘yes’ answer), low (1 = 1 positive answer), medium (2 = 2 or 3 positive answers) and high (3 = 4 or 5 positive answers)). The total score of all statements was categorised at mid-point into high adaptive capacity and low adaptive capacity.

*Explanatory variables*. We tested the statistical significance of 21 potential driving variables in explaining the distribution of perceived vulnerability between households (see Table 1). All of these have been frequently hypothesised in the literature as influencing disease vulnerability [16, 17, 22]. These included key household, community level and geographical factors that collectively shape households’ vulnerability/ adaptation to KFD. The specific indicators of the three dimensions of vulnerability (see Fig 1) were obtained from the literature and participants’ answers to questions about their lived experiences with KFD outbreaks and associated impacts of their livelihoods (Table 1). As illustrated in Fig 1, first, exposure was measured using two key indicators viz. the outbreak history (number of outbreak events reported between 2014 and 2019) of the area) and environmental factors (proportion of forest loss and elevation). Importantly, we assumed that exposure to KFD was not uniform given the spatial heterogeneity among households and individuals with regards to forest use and socio-cultural interactions within the landscape. We expected that KFD exposure may differ according to spatiality of households and individuals, with poorer and marginalised groups commonly living and working in more hazardous areas, such as inside the forest or carrying out activities such as wild honey extraction. Second, sensitivity was proxied by demographic and socioeconomic characteristics. Third, coping capacity was measured by five indicators viz. direct coping measures, human resource capability, economic capability, social capability and institutional capacity (see Fig 1, Table 1).

**Table 1:** Variables in perceived vulnerability and coping capacity in the regression model and a priori expectations.

Variables		Description of the indicators	Hypothesized relation
**Perceived vulnerability**		A binary indicator of household perception about contracting KFD: 1 if household is worried about contracting KFD; 0 if otherwise	
**Coping capacity**		A binary indicator of household coping capacity to KFD: 1 if household demonstrates capacity to adapt; 0 if otherwise	
Disease history	Outbreak history of area	Number of outbreaks (village) events reported from 2014 to 2018	+
Ecological Variables	Forest change	Proportion of forest loss	+
	Elevation	Elevation	+
Demographic variables	Age of Head (years)	Age (in years) of the household head	±
	Household Size	Current number of people recognised as household members	+
	Sex (gender)	A binary indicator of gender of household head: I if male-headed; 0 if female-headed	±
	Primary Education	1 if respondent has completed at least primary education; 0 if otherwise	±
	Family member involved in agriculture	Total number of household members directly involved in agriculture-based activity	+
	Cooking energy source	1 if household uses fuelwood as primary cooking energy source; 0 if otherwise	+
	Household sanitation	1 if household has access to a sanitary facility (toilet); 0 if otherwise	
	Social stratification (caste)	1 if household is classified as lower caste; 0 if otherwise	+
Livelihood activity	Forest-based activity	1 if household is engaged in forest activity; 0 if otherwise	+
	Agriculture-based activity	Total number of household members as plantation workers	+
Direct coping measures	KFD Vaccination history	1 if any household member have been vaccinated against KFD in the last 1 year; 0 if otherwise	
	Tick prevention measures	1 if household members adopt any measures to prevent tick bites; 0 if otherwise	
		1 if household members employ any kind of tick prevention measures on animals; 0 if otherwise	
Economic capability (Financial asset)	Alternative income source	1 if household has alternative sources of income; 0 if otherwise	
	Poverty status	1 if household is classified as ‘below poverty line’ (BPL); 0 if otherwise	+
Natural asset	Access to land	1 if household owns land; 0 if otherwise	
	Livestock holding	Household total livestock holding in tropical livestock units (proxy for household wealth)	±
Human resource capability (asset)	Household dependent ratio	Total number of dependents in household	+
	Disease Information access	A binary indicator of household’s access to information access (mobile phone ownership): 1 if household has access to a mobile phone(s); 0 if otherwise	
	Aggregate knowledge on KFD	A binary indicator of household’s knowledge about KFD: 1 if household has high level of knowledge about the disease (including prevention measures); 0 if otherwise	
Social capability (Social asset)	Socio-political status	1 if household head or member(s) has position within the local socio-political hierarchy	
Institutional capability (Physical factors)	Access to nearest PHC	Distance to nearest Primary Health Centre (PHC) (in kilometres)	-
	Access to nearest private hospital	Distance to nearest private hospital (kilometres)	-
	Proximity to forest reserve	Distance to nearest forest reserve (kilometres)	±
	Proximity to main road	Distance to nearest main road (kilometres)	+
**Location Effects**	Location (1 = Shivamogga)	1 if the household resides in Shivamogga district; 0 if otherwise	±

^1^The hypothesized influence of the variable on perceived vulnerability. Whereas a plus sign signifies a positive relationship is expected (i.e. a higher value of the variable is likely to increase vulnerability), a minus sign implies a negative relationship based on a priori expectation (i.e. likely lower vulnerability). Where both plus and minus signs are specified, it implies that no specific a priori expectations are made and the relationship is therefore subject to empirical investigation

*Outcome variables*. The perceived KFD vulnerability outcome variable could be one of two categories: (i) worried about contracting KFD (which includes those that stated that they were worried/ extremely worried, assigned a value of 1) or (ii) not worried about contracting KFD (which comprised those that stated they were either uncertain or not at all worried, assigned a value of 0). Likewise, households’ adaptation to KFD was specified as a dichotomous variable. We assigned the value of 1 for capacity of the household to adapt and zero in other cases. Vulnerability/ adaptation to KFD was empirically specified and modelled at the household level instead of the community level. This is premised on the assumption that major decisions regarding coping with KFD and associated stresses and livelihood processes (i.e. agriculture and forest-based activities and dependence) are taken at that level [16, 33]. Besides, exposure due to vector-borne diseases is inherently heterogeneous across landscapes due to the interaction of human ecology and resource use with complex ecology of vectors and hosts [15, 19]. Nevertheless, households are embedded within the wider community, which can significantly shape their decision-making processes in relation to allocation and use of particular resources [16].

*Univariate analysis*. Univariate binomial regression models were developed to study the association between of (1) socio-demographic, geographical location based factors with perceived vulnerability (vulnerability model), and (2) household demographic factors and location related factors with capacity to adapt (adaptation model) (see Table 1). Any explanatory variable associated with perceived vulnerability with a p-value <0.10 was selected for multiple mixed-effect logistic regression analysis. Collinearity among explanatory categorical variables was assessed by calculating Cramer’s phi-prime statistic. Thus a pair of variables were considered collinear if Cramer’s phi-prime statistic was >0.70, and one of each pair was excluded from the multivariate regression analyses.

*Multivariate analyses*. The explanatory variables with a univariate likelihood ratio chi-square or Fisher’s exact p-value of ≤ 0.1 were considered for integration into the initial multivariable model. Mixed effects logistic multiple-regression models (vulnerability and adaptation) were constructed using forward selection (likelihood ratio), step-wise approach. To account for spatial clustering, the variable study district was used as a random effects in the perceived vulnerability and adaptation model, respectively. The explanatory variables with a p-value of <0.05 were retained in the final model. Model adequacy of the final models with random effects was determined using the Hosmer-Lemenshow test, likelihood ratio chi-squared goodness-of-fit statistic and residuals. Results are presented as adjusted odds ratios (OR) and 95% CIs (see Table 1).

#### 4.4.2 In-depth key informant interviews

The interview data were transcribed and coded according to the emergent themes and topics, based on which key narratives were developed following Braun and Clarke’s [56] guide to thematic and content analysis. The themes were triangulated with the survey data and other secondary information collected based on which inferences and conclusions were drawn [4]. S1 Table outlines the key themes identified through the interviews and S2 Table identifies key informants interviewed (see supplementary data file).

## 5. Results

### 5.1 Households characteristics

Tables 1 and 2 summarise the results regarding household characteristics. Overall 68.6% of the households studied were male-headed, reflecting the social contexts in the region. The household size range between 1 and 7 persons in adult equivalent, with an average of 3 persons per household. The age of household heads ranged between 19 and 92 years, with an average of 54.28 years. Household-head refers to the senior-most member of the household who makes key decisions and whose authority is recognised among other household members [57]. Of the 229 surveyed households, only 27.9% household heads had secondary level education, with slightly more Wayanad participants (26.8%) than Shivamogga (26.8%) reporting same. 29.7% of participants had no education which was evenly distributed across the two study areas. Moreover, women household heads were less educated compared to their male counterparts. 16.7% of female participants reported they had attained secondary education relative to 33.1% of their male counterparts (χ^2^ value 6.637, p = 0.007). Concerning occupational activities, over half (56.8%) of the surveyed households were involved in agriculture-based activities, which were particularly pronounced in Shivamogga (67.5%) compared to Wayanad (33.3%). Off-farm income generating activities included daily wage labour, remittances and income from other sources. 71.6% of sampled households owned land, with an average land size of 0.323 and 0.199 acres reported in Shivamogga and Wayanad respectively. Altogether, significant proportion of surveyed households were classed as living ‘below poverty line’ (i.e. <Rs. 32 (£0.35) a day), reflective of the regional socio-demographic trends in Western Ghats region.

**Table 2:** Summary descriptive statistics for household socio-demographic characteristics.

Variables		Description of the indicators	Pooled (n = 229)			Shivamogga (n = 157)			Wayanad (n = 72)		
			n	%	Mean (SD)	n	%	Mean (SD)	n	%	Mean (SD)
**Perceived vulnerability**		Household worried about contracting KFD	101	44.1	-	65	41.4		36	50	-
		Household not at all worried about contracting KFD	128	55.9	-	92	58.6		36	50	-
**Coping capacity**		Household demonstrates capacity to adapt	34	14.8	-				1	98.6	-
		Household demonstrates no capacity to adapt	195	85.2	-				71	1.4	-
Disease history	Outbreak history of area	Number of outbreaks (village) events reported from 2014 to 2018(continuous)	**-**	**-**	12.32 (20.53)	**-**	**-**	11.46(21.05)	**-**	-	14.19(19.36)
Ecological Variables	Forest change	Proportion of forest loss (continuous)	**-**	**-**	0.398 (0.27)	**-**	**-**	0.41 (0.28)	**-**	**-**	0.37 (0.25)
	Elevation	Elevation (continuous)	**-**	**-**	691.86 (12.81)	**-**	**-**	674.8 (120.8)	**-**	**-**	726.9 (119.6)
Demographic variables	Age of Head (years)	Age (in years) of the household head continuous)	**-**	**-**	54.28 (13.85)	**-**	**-**	57.56 (13.77)	**-**	**-**	47.13 (11.14)
		15–24 years	2	0.9	-	1	0.6	-	1	1.4	-
		25–64 years	169	73.8	-	102	65		67	93.1	-
		65+ years (elderly)	58	25.3	-	54	34.4	-	4	5.6	-
	Household size	Current number of people recognised as household members (continuous)	-	-	3.86 (1.11)	-	-	3.70 (1.12)	-	-	4.19 (1.00)
	Sex (gender)	Women	72	31.4	-	39	25	-	33	46.8	-
		Men	157	69.6	-	118	75.2	-	39	52.2	-
	Education level	No formal education	68	29.7	-	48	30.6	-	20	27.8	-
		Primary	56	24.5	-	36	22.9	-	20	27.8	-
		Middle	25	10.9	-	20	12.7	-	5	6.9	-
		Matriculation/Secondary	45	19.7	-	33	21.0	-	12	16.7	-
		Higher secondary	19	8.3	-	9	5.7	-	10	13.9	-
		Technical Diploma	2	0.9	-	-	**-**	-	2	2.8	-
		Graduate and above	14	6.1	-	11	7.0	-	3	4.2	-
	Family member involved in agriculture	Total number of household members directly involved in agriculture-based activity (continuous)	-	-	1.64 (1.02)	-	**-**	1.87 (1.01)	**-**	**-**	1.14 (0.84)
	Social stratification (caste)	Scheduled caste	11	4.8	-	8	5.1	-	3	4.2	-
		Scheduled tribe	48	20.9	-	26	16.5	-	22	30.5	-
		Other Backward Caste (OBC)	134	58.5	-	117	74.5	-	17	23.6	-
		Other (Upper caste)	36	15.7	-	6	3.8	-	30	41.7	-
	Cooking energy source	Household uses fuelwood as primary cooking energy source	199	86.9	-	139	88.5	-	60	83.3	-
		Household has an alternative cooking energy source	30	13.1	-	18	11.5	-	12	16.7	-
	Household sanitation	Household has access to sanitary facility (toilet)	219	95.6	-	148	94.3	-	71	98.6	-
		Household without access to a sanitary facility	10	4.4	-	9	5.7	-	1	1.4	-
Livelihood activity	Occupation	Agriculture-based	130	56.8	-	106	67.5	-	24	33.3	-
		Unemployed	49	21.4	-	36	23	-	13	18.1	-
		Non-agriculture based	50	21.8	-	36	23		13	18.1	-
Direct coping measures	KFD Vaccination history	1 if any household member have been vaccinated against KFD in the last 1 year; 0 if otherwise	107	46.7	-	93	59.2	-	14	19.4	-
	Tick prevention measures	Household adopt measures to prevent tick bites	87	38.0	-	70	44.6	-	17	23.6	-
		Household does not adopt any measures to prevent tick bites	142	62.0	-	87	55.4		55	76.4	-
		Household members employ tick prevention measures on animals	80	34.9	--	64	40.8		16	22.2	-
		Household members do not employ any kind of tick prevention measures on animals	149	65.1	--	93	59.2	-	55	76.4	-
Economic capability (Financial asset)	Alternative income source	Household has alternative sources of income	82	35.8	-	76	48.4	-	6	8.3	-
		Household has no alternative income source	147	64.2	-	81	51.6	-	66	91.7	-
	Poverty status	Below poverty line (BPL)	185	80.8	-	139	88.5	-	46	63.9	-
		Above poverty line (APL)	44	19.2	-	18	11.5	-	26	36.1	
Natural asset	Access to land	Household owns land	164	71.6		106	67.5	-	58	80.6	-
		Household has no access to land	65	28.4		51	32.5		14	19.4	-
	Livestock holding	Household total livestock holding in tropical livestock units (proxy for household wealth) (continuous)	-	-	2.71 (3.36)	-	**-**	3.57 (3.65)	**-**	**-**	0.83 (1.33)
Human resource capability (asset)	Household dependent ratio	Total number of dependents in household	-	-	0.66 (0.83)	-	**-**	0.73 (0.87)	**-**	**-**	0.51 (0.71)
	Disease Information access	Household has access to a mobile phone	182	79.5	-	123	78.3	-	59	81.9	-
		Household has no access to a mobile phone	47	20.5	-	34	21.7	-	13	18.1	-
	knowledge on KFD	High (% with aggregate score of 4 or 5)	49	21.4	-	44	28.0	-	5	6.9	-
		Medium (% with aggregate score of 2 or 3)	62	27.1	-	49	31.2	-	13	18.1	
		Low (% with aggregate score of 1)	35	15.3	-	20	12.7	-	15	20.8	-
		Null (% with aggregate score of 0)	83	36.2	-	44	28.0	-	39	54.2	
Social capability (Social asset)	Socio-political status	Household head or member(s) has position within the local socio-political hierarchy	42	18.3	-	11	7.0	-	31	43.1	-
		No household members has position within the local socio-political hierarchy	187	81.7	-	146	93.0	-	41	56.9	-
Institutional capability (Physical factors)	Access to nearest PHC	Distance to nearest Primary Health Centre (PHC) (in kilometres) (continuous)	-	-	4.91 (3.47)	-	**-**	5.20 (3.71)	**-**	**-**	4.29 (2.80)
	Access to nearest private hospital	Distance to nearest private hospital (kilometres) (continuous)	-	-	11.20 (10.79)	-	**-**	11.55 (12.59)	**-**	**-**	10.44 (5.04)
	Proximity to forest reserve	Distance to nearest forest reserve (kilometres) (continuous)	-	-	3.71 (6.53)	-	-	1.60 (2.58)	-	-	8.31 (9.53)
	Proximity to main road	Distance to nearest main road (kilometres) (continuous)	-	-	1.51 (8.02)	-	**-**	1.17 (1.63)	**-**	**-**	2.28 (14.14)
Location	Location	Household resides in Shivamogga district	157	68.6	-	-	**-**	**-**	**-**	**-**	**-**
		Household resides in Wayanad	72	31.4	-	-	**-**		**-**	**-**	**-**

### 5.2 Past KFD exposure, knowledge and adaptation pathways

**Fig 4:**
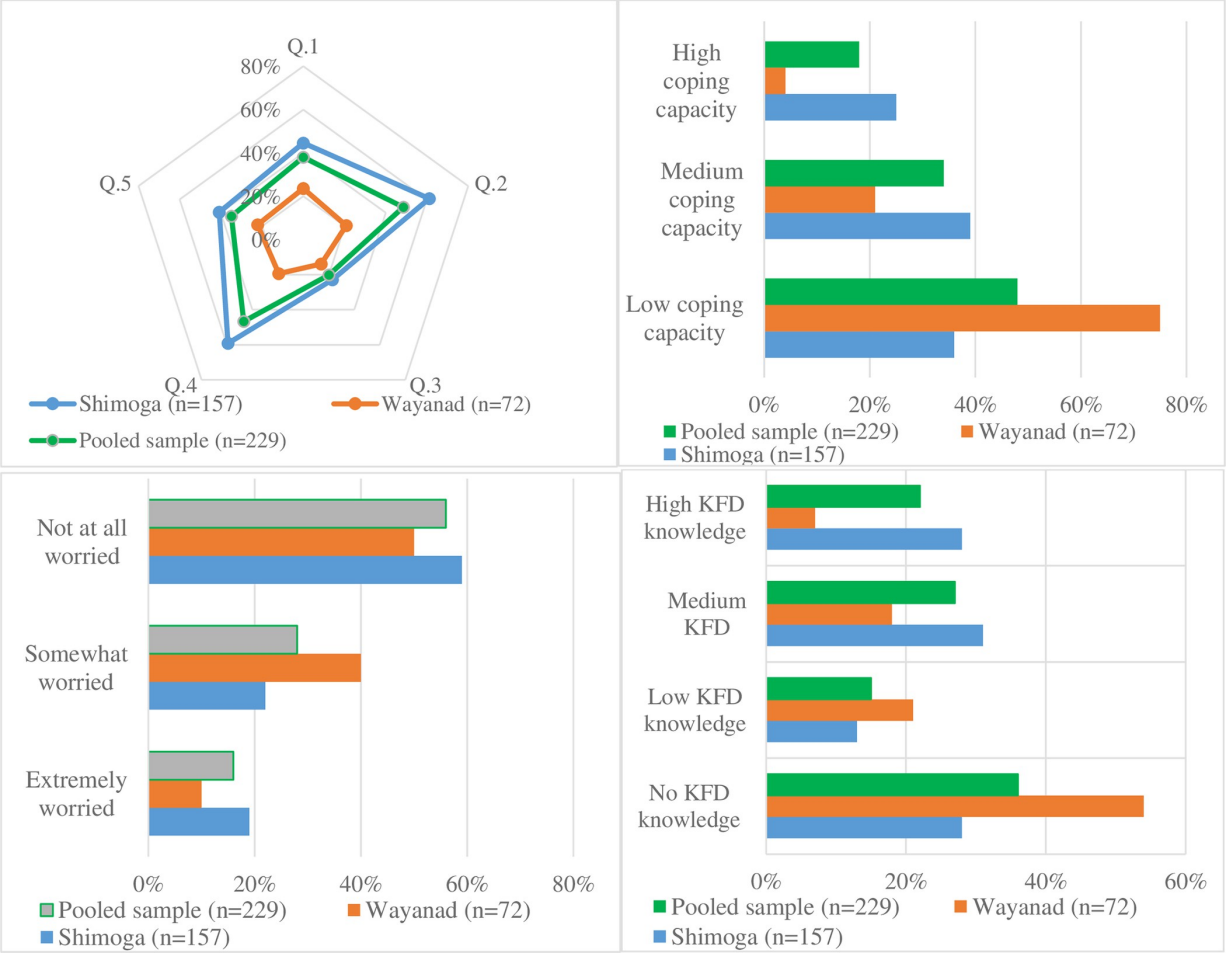
Assessment of households KFD knowledge and adaptation based on survey questionnaire. **(A)** Adoption of KFD prevention measures. [(Q.1) Do you adopt any measures to prevent tick bites on your body? (Yes/No), (Q.2) Do you know of any prevention measures for KFD? (Yes/No), (Q.3) Do you know what to measures to take if you suspect you have contracted KFD? (Yes/No), (Q.4) Have you been vaccinated against KFD? (Yes/No), and (Q5) Do you employ any kind of tick prevention measures on your animals? (Yes/No)]; **(B)** Household coping capacity to KFD. [1 = low coping capacity, 2 = medium coping capacity, and 3 = high coping capacity]; **(C)** Worried about contracting KFD, **(D)** Composite score of KFD knowledge. [0 = no knowledge about KFD, 1 = low KFD knowledge, 2 = medium knowledge, 3 = high KFD knowledge].

Regarding perceptions of KFD (Table 3, Fig 4), approximately 44% of the participants (n = 101) reported being worried about contracting KFD, with less than one-third (26.6%) of participants claiming they had previously contracted or knew someone with KFD. There was no significant variation across social groups or study areas. A slightly higher proportion of Shivamogga households (54.8%) than Wayanad (40.3%) acknowledged that people are likely to contract KFD, which is not very surprising given KFD endemicity in the former. Likewise, approximately half of households (51.1%) acknowledged that the propensity of KFD exposure is linked to their forest use, with nearly three-quarters of Shivamogga households (71.3%) relative to Wayanad (6.9%) responding in the affirmative. A further cross-tabulation analysis showed that 41.1% of the pooled sample who indicated they had good knowledge of different KFD risk factors also perceived KFD as severe (χ^2^ value 34.26, p<0.001), which is evenly distributed across the two study areas. Altogether, the above results underscore the importance of disease knowledge in shaping vulnerability as highlighted in by a Wayanad tribal chief in an interview:

*“I admit that we lack the knowledge*, *but to my understanding*, *most of the things we can do to avoid getting KFD is by observing nature…We scheduled tribes are listed as high risk people as our livelihood activities and forest caring is different from other people*. *The other people who always wear full dress with big boots like the white people [reference to our field team who undertook ecological sampling of KFD vectors] are never exposed to this disease*.*”* (WD-2, Wayanad)

**Table 3:** KFD knowledge and perceptions by study region.

Categories	Study Regions		Full sample (N = 229)
	Shivamogga (n = 157)	Wayanad (n = 72)	
**Past history with KFD**			
Have ever had/contracted KFD	10 (6.4%)	7 (9.7%)	17 (7.4%)
Know someone with KFD	28 (17.8%)	16 (22.2%)	44 (19.2%)
Death of family KFD survivor	3 (1.9%)	1 (1.4%)	4 (1.7%)
Never heard of KFD/ Monkey fever	38 (24.2%)	25 (34.7%)	63 (27.5%)
**Risk perceptions**			
High perceived severity of KFD	89 (56.7%)	2 (2.8%)	91 (39.7%)
KFD as a major health issue in the region	110 (70.1%)	49 (68.1%)	159 (69.4%)
KFD not at all significant health issue in the region	47 (29.9%)	23 (31.9%)	70 (30.6%)
People are likely to contract KFD	86 (54.8%)	29 (40.3%)	115 (50.2%)

Regarding implementation of adaptive measures to reduce perceived KFD vulnerability or accommodate long-term shifts in livelihood activities, participants were asked whether they adopted any preventive practices as evidenced in Fig 4A. Based on a three-point Likert scale measurement, an overwhelming majority of households (85.2%) reported low coping capacity to KFD, which was quite evenly distributed across the two study sites. The most common coping strategies adopted by households to lower their vulnerability were vaccination, application of DMP oil and forest avoidance [4]. Nevertheless, the perceived effectiveness of these strategies as reported during the interviews was very low across both study areas, with several participants asserting that their coping strategies to minimise KFD exposure were not effective. For instance, the survey data suggest a generally low vaccination coverage (46.7%) in the study areas, with less than one-third (19%) of Wayanad households relative to their Shivamogga counterparts reporting been vaccinated (Fig 4A). Household adoption of KFD preventive measures was positively correlated with poverty status (χ^2^ value 13.91, p<0.001), with more poor households (88.9%) relative to non-poor (11.1%) implementing preventive measures. Likewise, the majority of lower caste households (65.3%) as against upper caste ones (25%) adopted KFD preventive measures (χ^2^ value 20.35, p<0.001), and more households worried about contracting KFD (72.3%) versus unworried households (48.4%) adopted recommended preventive practices (χ^2^ value 13.26, p<0.001). The dynamics of disease information and adaptive capacity in the focal study areas has been reported elsewhere [4]. Livelihood diversification is another important factor associated with disease sensitivity. Male-headed households had more opportunities and elaborate portfolios of more income in the non-agricultural economy, including casual construction and driving jobs. The combination of different adaptive strategies, information sources and livelihood options among households influenced their sensitivity to KFD. Nevertheless, avenues to reduce sensitivity to KFD-related impacts across the surveyed villages remain limited, prompting feelings of frustration and disenfranchisement over forest restrictions which created livelihood uncertainties. The reflections of two KFD-survivors (a plantation worker and a house wife) during separate interviews in Shivamogga are illustrative:

*“We go to the plantation as it is our regular work*. *We need to go to the plantation*, *it is necessary for us*. *My brother goes to the plantation*, *he collects the dry leaves and woods*. *We cannot do anything*. *Everything [livelihood] is from the plantation*! (SA-4, Shivamogga)

*“No*, *we should be allowed to go to the forest*. *We cannot do anything without it [in reference to collecting firewood and wild honey]*. *We have to work*. *We can’t live without work*!*”*(SA-6, Shivamogga)

### 5.3 Factors influencing perceived vulnerability to KFD

Table 4 presents our regression results which explore the determinants of KFD vulnerability along individual and household lines. The dependent variable in this context is binary variable reporting worried about contracting KFD or demonstrating capacity to cope and the models are estimated using logistic regression. The first three set of columns correspond to perceived vulnerability, while the fourth, fifth and sixth set of columns relate to coping capacity. For vulnerability, we regress “worried about contracting KFD” with individual, household and community level factors controlling for regional level fixed effects. In the case of adaptation, we regress “ability/capacity to cope” with individual, household and community level factors and also control for region-level fixed factors.

**Table 4:** Multivariate analysis of factors influencing household perceived vulnerability and adaptation to KFD in Shivamogga and Wayanad. In interpreting the results, we use the coefficient and Adjusted Odds Ratios (AOR), SE = Standard Error; CI = Confidence Interval values, and P values.

Variable	Perceived KFD vulnerability				Coping Capacity			
	Coeff.	S.E.	AOR [95%CI]	P-value	Coeff.	S.E.	AOR [95%CI]	P value
**KFD Outbreak History**								
Log total number of KFD outbreaks (2014–2018)	-0.220	0.266	0.802 [0.477–1.351]	0.407	-0.034	0.271	0.967 [0.569–1.643]	0.900
**Sensitivity Factors**								
***Demographic Characteristics***								
Age of Household Head (years)	0.037***	0.016	1.038 [1.006–1.071]	0.019	-0.013	0.016	0.987 [0.956–1.019]	0.415
Household Size	0.380**	0.199	1.462 [0.989–2.160]	0.057	0.158	0.203	1.172 [0.787–1.745]	0.436
Gender of Household Head (1 = Male)	0.152	0.396	1.164 [1.164–0.535]	0.701	-0.301	0.421	0.740 [0.324–1.688]	0.475
Completed at least primary education (relative to none)	0.272	0.418	1.312 [0.579–2.974]	0.516	-0.145	0.439	0.865 [0.366–2.045]	0.742
Cooking energy source	0.711	0.572	2.035 [0.663–6.244]	0.214	-0.187	0.595	0.830 [0.258–2.666]	0.754
Household sanitation	2.242	1.403	9.410 [0.602–147.134]	0.110	1.475	1.056	4.370 [0.551–34.642]	0.163
Social stratification (caste)	-0.650	0.547	0.522 [0.179–1.524]	0.234	-0.381	0.603	0.684 [0.210–2.226]	0.528
***Livelihood Activity***								
Number of HH members engaged in forest activity	0.984**	0.434	2.675 [1.142–6.264]	0.023	0.855**	0.399	2.352 [1.077–5.139]	0.032
Number of HH members engaged in agriculture activity	0.302	0.185	1.353 [0.941–1.946]	0.103	0.391**	0.204	1.478 [0.991–2.206]	0.056
**Coping Capacity Factors**								
***Direct Coping Measures***								
KFD vaccinated	1.122***	0.419	3.072 [1.352–6.979]	0.007	-	-	-	
Adoption of tick prevention measures (on body)	0.726*	0.393	2.067 [0.957–4.465]	0.065	-	-	-	
Adoption of tick prevention measures (on animals)	0.374	0.388	1.453 [0.679–3.107]	0.335	-	-	-	
***Economic capability (Financial Assets)***								
Alternative income source	-0.686	0.437	0.504 [0.214–1.186]	0.116	-0.099	0.451	0.906 [0.374–2.194]	0.827
Poverty status of household	-1.373***	0.508	0.253 [0.094–0.685]	0.007	-0.110	0.519	0.896 [0.324–2.479]	0.833
***Natural Assets***								
Access to land	-0.986**	0.458	0.373 [0.152–0.916]	0.032	-0.370	0.440	0.691 [0.292–1.637]	0.401
Log total livestock holding	-0.314	0.643	0.731 [0.207–2.576]	0.626	1.443***	0.579	4.233 [1.361–13.167]	0.013
***Human Resource Capability (Assets)***								
Log household dependency ratio	-0.631	1.088	0.532 [0.063–4.488]	0.562	0.004	1.110	1.004 [0.114–8.850]	0.997
Disease information access (phone ownership)	0.264	0.446	1.302 [0.543–3.120]	0.554	1.046**	0.465	2.846 [1.145–7.076]	0.024
Aggregate knowledge on KFD	0.378	0.448	1.460 [0.607–3.509]	0.398	2.769***	0.590	15.950 [5.023–50.651]	0.000
***Social capability (Social Asset)***								
Socio-political status of household	-0.148	0.496	0.863 [0.327–2.280]	0.766	-0.006	0.541	0.994 [0.344–2.870]	0.991
***Institutional capability (Physical factors)***								
Log access to nearest public health centre (PHC)	0.673	0.503	1.960 [0.732–5.252]	0.181	0.170	0.552	1.185 [0.402–3.497]	0.759
Log access to nearest private hospital	1.188**	0.505	3.281 [1.220–8.820]	0.019	-0.692	0.508	0.500 [0.185–1.354]	0.173
Log proximity to main road	0.763***	0.290	2.144 [1.215–3.783]	0.008	0.269	0.261	1.309 [0.785–2.184]	0.302
**Location Characteristics**								
Study Region (1 = Kakum)	-1.485***	0.606	0.226 [0.690–0.743]	0.014	0.910*	0.599	2.485 [0.768–8.041]	0.129
Constant	-6.339*	2.022	0.002	0.002	-3.026*	1.781	0.048	0.089
	-2log-likelihood = 237.350				-2log-likelihood = 218.637			
	Nagelkerke R Square = 0.895				Nagelkerke R Square = 0.466			
	Model Chi-Square = 76.920***				Model Chi-Square = 98.470 ***			

* Significant at p < 0.10

** Significant at p <0.05

*** Significant at p<0.001

Significant determinants of household perceived vulnerability included age of household head, household size, number of members engaged in forest activity, vaccination status, adoption of tick ***prevention*** measures, poverty status, access to land, proximity to private hospital and main road and location (Table 4). This suggests that both intra and extra-household factors operate to shape households’ perceived vulnerability in the study contexts. Concerning demographic factors, household age and household size both exerted a significant positive effect on perceived vulnerability. For example, households headed by elderly persons tended to be more worried about contracting KFD compared with younger persons. To the extent that elderly household heads have limited agency/capacity to support their families against KFD, impacts rendered them more vulnerable. Similarly, the larger a household the more likely for it to be vulnerable since a larger number of household members could potentially be exposed to the disease. Indeed, an additional member of a household increases the odds of being worried about contracting KFD by 46.2% (AOR: 1.462, 95% CI: 0.989–2.160). As expected a priori, the higher the proportion of household members engaged in forest-related activities the more likely for it to be vulnerable since KFD is an ecotonal disease [15] (AOR: 2.675, 95% CI: 1.142–6.264). The results showed no statistical differences between males and females in terms of their perceived vulnerability. Likewise, no differences were detected between literate and illiterate-headed households in perceived vulnerability.

Vaccination status was positive associated with perceived vulnerability suggesting that self-reported vaccinated households are more likely to worry about contracting KFD. For instance, for every additional household member vaccinated the odds of being worried about contracting KFD increases by 207.2% (AOR: 3.072, 95% CI: 1.352–6.979). Likewise, households in which a higher proportion of members adopted tick prevention measures tended to perceive themselves as vulnerable (AOR: 2.067, 95% CI: 0.957–4.465). Given that interventions for KFD are limited in their effectiveness [11, 58], it follows that the adoption of coping measures (including vaccination) may not necessarily assuage worry about contracting it. This assertion was further corroborated by some interviewees (belonging to tribal groups) who sceptically remarked “*we have doubts about the (vaccination and application of DMP oil) measures which government is glorifying*” (WD3, Wayanad), implicitly echoing a sense of distrust over government-sponsored vaccination interventions [4, 11]. At the same time other interviewees argued that taking the recommended three vaccine doses in combination with other personal tick prevention measures (such as applying DMP oil and wearing long clothing) affords protection against KFD, particularly amongst highly vulnerable groups. Two contrasting views by a female plantation worker (KFD-survivor) and a male forest watcher in Shivamogga and Wayanad respectively are instructive:

*“…Some (in reference to neighbours) said that there is an injection [vaccine] for KFD which we had taken in two months in the beginning*. *KFD had come to us even after two doses*. *Though*, *I had taken the third dose recently*.*”* (SA- 5, Shivamogga)

*“I don’t think the vaccine itself will protect me*. *So I use other methods (like wearing gumboots*, *neem oil and proper body check) whichever is feasible to prevent tick bites*.*”* (WD-3, Wayanad)

The poverty status variable exerts a significant negative effect on perceived vulnerability suggesting that Below Poverty Line households are less likely to worry about contracting KFD relative to Above Poverty Line households (AOR: 0.253, 95% CI: 0.0094–0.685). This finding is contradictory to both theory and *a priori* expectation that poorer households’ are more likely to be vulnerable due to their limited capabilities or agency [4, 33]. We hypothesise that this could be suggestive of the interplay of other factors (e.g. disease knowledge and religio-cultural beliefs) that might have imbued confidence (or hopefulness) in their ability to cope (through survival skill learning) with the disease. Indeed, some participants particularly those of scheduled tribe or caste backgrounds despite their limited agency shared that experience of KFD is a catalyst for long-term adaptation planning. Reminiscing about the previous KFD outbreak, a 75-year old tribal chief in Wayanad highlighted that *“it [reference to KFD] did not teach us a bad lesson*, *but it gives us an opportunity to rethink where we lost our knowledge about the forest and we have to observe natural changes and should be able to predict the impact of it*.*”* (WD-2, Wayanad). Corroborating this assertion was another participant who was working as a “tribal promoter” (a community development worker under the state government’s tribal welfare program) from a *Kattunayakan* village (in Wayanad), reflecting on the KFD impacts, argued that *“education makes people comfortable about the disease”*. He further remarked:

*“Yeah I agree that people panic about the disease*, *but clear awareness programs starting from life cycle of ticks to how each of our (Kattunayakan) activities both livelihood and other traditional effects*, *where it is hit*, *and how to tackle this without losing anything*.*”*(WD-1, Wayanad)

Moreover, access to land (proxy for physical asset) is negatively related to perceived vulnerability implying that households owning land are less likely to be vulnerable. For example, an additional acre of land owned by a household lowers the odds of being worried about contracting KFD by 62.7%. Consistent with *a priori* expectation, the coefficient of the distance to main road and private hospital variables (the two geographical factors) are positive and statistically significant at the 1 and 5% respectively. For instance, an additional kilometre in distance from a private health facility increases the odds of being worried about contracting KFD by 228.1% (AOR: 3.281, 95% CI: 1.220–8.820). This finding may not be surprising given the remoteness of the majority of surveyed households from primary healthcare infrastructure in the study areas. Moreover, the location variable was inversely associated with perceived vulnerability implying that surveyed households situated in Shivamogga are less likely to be worried about contracting KFD relative to Wayanad households. The locational differences may be suggestive of some degree of complacency of households situated in Shivamogga given the long history of KFD in the area.

### 5.4 Determinants of households’ adaptive capacity

Factors influencing households’ capacity to implement adaptive measures to KFD and its potential impacts are summarised in Table 4. The significant predictors of household capacity to adapt included livelihood activity, livestock holding, phone ownership, KFD knowledge and location. Contrary to *a priori* expectations, the higher the proportion of household members engaged in forest/agricultural activities, the more likely it is for that household to report being able to cope with KFD (AOR: 2.352, 95% CI: 1.077–5.139). Evidence from empirical literature highlights frontline forest staff (such as forest watchers) and plantation workers as being highly susceptible to infected tick bites due to the nature of their occupations [15]. The livestock holding and phone ownership (proxy for access to disease information) variables both exert a significant positive influence on capacity to cope, which is consistent with theory and *a priori* expectation. For instance, an additional unit of phone owned by a household increases the odds of coping with KFD by 184.6% (AOR: 2.846, 95% CI: 1.145–7.076). This concurs with a similar observation made by Asaaga et al. [4] that smallholders valued disease information as critical to their adaptation to KFD in the Western Ghats region. Likewise, the coefficient of the level of knowledge variable exerts a significant positive effect on adaptive capacity (AOR: 15.950, 95% CI: 5.023–50.651), implying that knowledge through previous disease experience could be an important ‘intangible’ resource for adapting to KFD and its associated impacts. In support of the descriptive analysis (Section 5.2), survey respondents who demonstrated a high level of knowledge about KFD transmission pathways and preventive measures tended to have a higher propensity to implement adaptive response to KFD-related impacts relative to their counterparts with limited knowledge. Furthermore, the location variable exerts a positive significant influence on adaptive capacity indicating that surveyed households situated in Shivamogga are more likely to adapt compared with Wayanad households. For instance, for every additional household in Shivamogga the odds of coping with KFD increases by 148.5% (AOR: 2.485, 95% CI: 0.768–8.041). To the extent that experience with KFD is an important catalyst for long-term adaptation, then it is not surprising that Shivamogga households relative to their Wayanad counterparts are better positioned to successfully adapt to the disease and as a consequence to be less concerned. During the qualitative interviews, several participants particularly in Wayanad were more uncertain about KFD and its associated impacts which lends further credence to the above observation. Two typical views in this regard were given by a tribal leader and an Accredited Social Health Activist (ASHA) worker in Wayanad:

*“When we talk about KFD*, *as it is new disease to us*, *it brought the uncertainty*, *about our hamlet*, *activities*, *our medicinal practice*, *our health system*, *so that made us feel bad always*.*”* (WD-1, Tribal Promoter, Wayanad)

*“When I attended the last meeting the officials were talking even the forest entry ban (when 2 death cases reported last week*, *government banned forest usage completely) it is like cutting their [Kattunayakan] cultural roots*.*”* (ASHA worker–Wayanad)

Our findings indicate that vulnerability is contextual and differentially experienced by individual households and groups (e.g. land-poor and lower-caste) with different capacities to implement strategies in response to disease consequences, even though the majority of surveyed households are impoverished.

## 6. Discussion

With the intensified calls for contextually appropriate and targeted interventions (partly instigated by the COVID-19 pandemic) to tackle emerging disease risks in geographical hotspots, existing efforts are often hampered by the poor understanding of underlying socio-spatial drivers of disease vulnerability and adaptive capacity [4, 17]. Existing scholarship has largely problematized diseases from a biomedical perspective overlooking the interplay of complex biosocial dynamics that underpin disease vulnerability and adaptive capacity within and across socio-spatial contexts [4, 42]. In this context, perceived vulnerability has been increasingly viewed as the closest proxy for actual disease vulnerability as affected households themselves are understood to make their health-seeking and adaptation decisions based on their subjective perceptions of their vulnerability to disease risks and associated impacts [59, 60]. Consistent with most empirical studies on vulnerability [16, 21, 22], we thus operationalised perceived disease vulnerability as households’ subjective estimates of the extent to which they were worried about KFD and its associated impacts in the study regions.

We detected important social differences in patterns of perceived disease vulnerability that need to be considered to better inform interventions at the local level. Our findings provide insights into how disease interventions can be effectively tailored and operationalised to optimise health and livelihood outcomes within the limits of local health systems in India. We discuss two key implications of this study: first, who perceives themselves as vulnerable and why and what does this mean for local adaptive capacity; and second, to what extent can a focus on social vulnerability help national and international health planners improve health interventions and prioritise among neglected endemic zoonotic diseases?

### 6.1 Who is most vulnerable and why?–Unpacking social differences in perceived KFD vulnerability

Conventional analyses aimed at informing disease interventions are often confronted with a practical trade-off of treating ‘smallholders’ as a homogenous group in comparison to other “at risk” populations and describing the complexities of relations between and within social groups [4]. Several studies highlight the drawback of ‘blanket’ characterisation of smallholders as ‘vulnerable’ in informing effective and targeted interventions, arguing that social differences particularly within groups can help avoid (unintentionally) reinforcing existing inequalities [17, 19]. Consistent with this argument, our findings underscore that perceived disease vulnerability is socially differentiated. Our existing disease control frameworks need to be broadened to better capture underlying social attributes and circumstances that cause vulnerabilities and how differences in capabilities (i.e. why some are able to cope with problems when others cannot) can inform targeted disease prevention/ adaptation pathways. Our data suggest that the groups that perceive themselves as most vulnerable to KFD-related impacts were the poor: land poor, labour-poor and information-poor. These households are particularly prone to greater uncertainties in livelihood options and most susceptible to poor health as they occupied ‘spaces of vulnerability’ shaped by historical processes of resource contestation and exclusion [25].

Importantly, the differences in the lived experiences, priorities and perceptions of smallholder and tribal households can have far-reaching implications for the typology of interventions and adaptation strategies that are contextually feasible and appropriate. Whereas human vaccination may be broadly beneficial as a conventional disease control response, they may in fact benefit from additional coping strategies such as providing tailored information on risky practices and making health extension support more available to remote families residing in the forest [4, 42, 52]. As evidenced in sub-Section 5.3, vaccinated households are more likely to perceive themselves as vulnerable which suggests the need for broader set of interventions (beyond vaccination) given longstanding mistrust and deep-seated concerns about efficiency of the existing KFD vaccine [4, 11, 58]. This observation lends credence to the argument that addressing poverty and other socio-economic inequalities among forest-based communities is critical to bolstering local adaptive capacity [4, 17, 52, 61]. Moreover, our results that poorer households are less likely to be worried about contracting KFD (not necessarily due to uptake of adaptive strategies) may indicate maladaptation effect based on an underlying worldview of persistence and absorption of ‘adversity’ in hope of fashioning an adaptive response through ‘survival skill learning’ in the context of poverty and inequality [33, 62].

To the extent that interventions are geared towards alleviating disease impacts, the similarities and differences identified in this study also echo deep-seated social inequities that underpin perceived disease vulnerability and adaptation. It therefore follows that homogenous characterisation of smallholders as ‘vulnerable’ could compromise or operate to favour certain intervention pathways, which might threaten or jeopardise the already ‘precarious’ livelihoods of certain social groups with weaker bargaining power. A case in point is the blanket imposition of forest bans which has meant that certain forest-dwelling tribal groups (e.g. *Kattunayakan*), with limited agency and bargaining power, have had to switch to other unstable alternatives (e.g. non-egalitarian lifestyles) or resort to ‘riskier’ livelihood options. This further echoes differences in the context of marginality which operate to limit coping capacity of affected households. As Yaro [34] argued, shrinking assets and reduction of access (in this case forest resources) by a large segment of the “at-risk” population reduces entitlements (e.g. cultural capital) and further entrenches and exacerbates existing inequalities.

Even within the same socio-spatial context, certain sub-groups (particularly elderly-headed households, lower caste households, ‘information-poor’ households) face greater disadvantage in negotiating/ leveraging available opportunities for successful adaptation pathways. As evidenced in section 5.3, larger households for instance are susceptible to KFD exposure probably because there is a higher likelihood to capture a forest-related activity, which may increase the risk of human-tick contact. It is thus essential that the dynamics of socially-differentiated vulnerability (structuring poverty and marginality) and the underlying historical context of forest exclusions is specifically acknowledged and prioritised in the KFD system. To the extent that disease control efforts fail to recognise or prioritise the heterogeneity of ‘at risk’ groups and their socio-cultural and institutional contexts, then they risk ‘half-baked’ interventions that lack the propensity to be transformative and galvanise local adaptive capacity. Away from intervention-orientated analyses, there is a need to focus attention on the deeper processes of exclusion and social inequalities that undergird perceived disease vulnerability [17, 59].

### 6.2 Social vulnerability a useful construct for improving disease interventions?

While conventional and often coarse-scale biomedical analyses, may yield general insights about “at-risk” populations, they often lack granular scale understandings of the diversity between social groups and the dynamics of their local environments that embody their lived realities and experiences. It therefore follows that nuanced bio-cultural understandings of the varied perceptions and experiences within and across focal groups may help bridge the fundamental disconnect between biomedical understandings of ‘risks’ and livelihood impacts of disease events at the local and landscape levels. As such, focussing on (multi-dimensional) vulnerability in Fig 1 afforded a useful analytical lens for a nuanced interrogation of household experiences and perceptions critical to understanding exposure to emerging disease risks or multiple environmental stressors. We therefore concur with Mangesho et al. [59], who argued that appreciating how disease risks are perceived at the local level is crucial to anticipating the livelihood and welfare impacts, given that households’ response to disease risks is predicated on their experiences and capabilities [4, 61].

An emerging body of literature have used social vulnerability to explore these dynamics [4, 17, 19, 59]. As highlighted in earlier sections, perceptions of disease reveal who is most affected by disease events, coping strategies and serves as a useful indicator of patterns of (uneven) exposure. Interviewees highlighted drawbacks of interventions around forest avoidance and switches to uncertain livelihood activities as disadvantageous to their welfare, which fail to recognise the intricate web of relations between local communities and forest environments (see Section 5.3). Whereas livelihood diversification is often framed as positive response to risk, through spreading of risk, it may not necessarily culminate in greater resilience to future disease risks. Our findings suggest that what is needed for meaningful adaptation is targeted long-term policy support considering the structural issues relating to smallholders agency and resource-use arrangements. Through a contextual ‘place-based’ vulnerability analyses at the local and landscape scale, differences in capabilities and conditions underpinning individual households’ susceptibility to disease-related shocks and impacts can be holistically understood and appropriate interventions formulated. Moreover, a social vulnerability framework could help elucidate the interplay of household entitlements and agency by capturing the variety of tangible (resources) and less tangible factors (perceptions of self and opportunities to implement changes in one’s life) that support transformative adaptation to emerging disease risks.

This study has some limitations that should be considered when interpreting the results. First, the cross-sectional nature of our study suggests that we cannot imply causality in our findings. Nevertheless, our findings present information on potential determinants of perceived KFD vulnerability, which can be adopted in prospective observational studies to understand the causal relationship between these potential determinants and KFD vulnerability. Secondly, the analysis is based on a self-reported measure of perceived KFD vulnerability. Self-reported measures of perceived KFD vulnerability may reflect actual vulnerability but could also be influenced by several other factors, including recall bias and systematic changes in a respondent’s understanding and lived experience of what makes one vulnerable [4]. Whereas obtaining actual measures of KFD vulnerability is difficult, attempts should be made to at least validate self-reported measures. Thirdly, to the extent that exposure to KFD may lead to a modification of households’ lifestyles over time, the analysis of one-year of cross-sectional data may not fully capture the scope of adaptation behaviours and practices. It therefore follows that the extent of households’ perceived vulnerability may be greater or lesser than the actual impact. Also related to data limitations, we could not fully unpack the underlying power relations within and across groups important for an integrated understanding of the political ecology of KFD dynamics. We suggest future studies explore in detail the contours of power relations and the interplay with disease vulnerability [17]. Besides, relevant vulnerability indicators such as income levels and housing conditions, local health workers per head of population, among others, were not available in a spatially disaggregated format. Nevertheless, the 21 vulnerability indicators considered are those often commonly identified in the literature [16, 17, 22]. Fourth, the relatively small sample size used in this study and the focus on only two of the KFD-affected districts may limit the extrapolation of the results to all KFD-affected populations in the Western Ghats region. Moreover, the field team’s positionality as ‘insiders’ (i.e. have lived or worked in study districts) might have influenced their comprehension of the research problem and by extension selection and approach of the study contexts. In this light, the researchers (MR, SDK, TS and PNS) ability to fluently communicate and relate with the local traditions played a pivotal role in facilitating access to the studied communities. Whereas this was useful in brokering access to respondents at face-value, it could also present some negative ramifications in terms of anticipation of future support (although it was made clear at the outset of the interviews that no direct benefits would accrue). Some respondents for instance suggested that the field team by virtue of their nativity could easily relate with and better capture their stories to the relevant authorities. Thus to avert any negative reflexivity issues and minimise bias, we employed a number of precautionary strategies such as repeated assurance of study objectives, use of pseudonyms and team debriefing. We however admit that the researchers’ identity and personal orientation could have also ‘coloured’ or ‘blurred’ their attention to some otherwise promising lines of enquiry (i.e. discovery of new concepts) as they may already be familiar with some of the observations made by respondents. Finally, the combination of qualitative and survey data afforded nuanced explanations regarding differential perceptions of household vulnerability and adaptation which otherwise would have been masked in a wholly quantitative investigation.

## 7. Conclusion

Understanding social differences in patterns of disease vulnerability is necessary for policymakers to develop contextually sensitive control and adaptation interventions for long-term resilience [16, 17]. Yet, there is a relative dearth of empirical focus on underlying social vulnerabilities (why some individuals get infected and not others) and adaptation needs of different ‘at-risk’ groups with respect to neglected zoonotic diseases [4, 17, 19]. This has often meant an over-emphasis on the biophysical factors in the emerging scholarship ignoring a range of contextual issues that shape individuals and households’ exposure, sensitivity, and adaptive capacity. This conceptualisation overlooks the role of individual households’ lived experiences and perceptions in (re-)negotiating new avenues and strategies to cope and adapt to disease risks in their everyday lives as well as how risk compounding or entrenching the factors that undergird households’ vulnerabilities [19, 59, 61].

The present study lends empirical support to the notion that policy prescriptions need to move beyond purely biomedical or techno-managerial solutions to disease risks and prioritise underlying social, political, and economic factors that influence the agency and capacity of individuals and households to implement or capitalise on opportunities to successfully adapt. Importantly, the findings highlight heterogeneity amongst individual households in terms of socio-demographic backgrounds, wealth status, information access and brokerage etc. Homogenised discourses and interventions centred on ‘smallholder’ vulnerability risk neglecting important policy-relevant distinctions, leading to skewed or sub-optimal solutions, and could overlook critical social drivers of inequality and poverty undergirding vulnerability. This study has further demonstrated the importance of nuanced characterisation of ‘vulnerable’ groups in a way that sufficiently accounts for heterogeneity. For example, policymakers and implementers should see dichotomies like poor versus non-poor, land-owning versus landless, informed versus ill-informed not as fixed and mutually exclusive categories but as constantly transforming positions on interacting and overlapping scales depending on the prevailing social and economic contexts [17, 19]. In future, studies assessing household-level vulnerability to KFD-related impacts should explore the use of panel data as well as other participatory approaches (e.g. community risk and knowledge mapping) to understand additional socio-spatial nuances around adaptation strategies, empowerment and intervention requirements of specific vulnerable sub-groups to bolster local adaptive capacity.

In summary, as an assemblage of social, political, economic and biophysical processes operating at the micro and macro scales to shape households’ KFD vulnerability, inclusive policies and interventions prioritising empowerment, supporting health extension services and creating alternative income options and increasing access to tailored disease information are critical in engineering transformative adaptation and resilience of different ‘at-risk’ groups to KFD and other emerging disease risks. This underscores the need for broader stakeholder engagement within a multi-dimensional vulnerability framework to better understand, prioritise and respond to emerging neglected zoonotic disease risks.

## Supporting information

S1 Checklist Inclusivity in global research questionnaire. (DOCX)

S1 Table Main thematic analysis results summarises based on key informant interviews with KFD survivors, district and taluka managers regarding their experiences and perceptions about 2018/19 KFD outbreak in the Western Ghats area of India. (DOCX)

S2 Table Key informant interviews. (DOCX)
