# Supplementary material for: The role of social vulnerability in improving interventions for neglected zoonotic diseases: The example of Kyasanur Forest Disease in India
Source: PLOS Glob Public Health. 2023 Feb 8;3(2):e0000758. doi: 10.1371/journal.pgph.0000758 (PMC10021172; doi:10.1371/journal.pgph.0000758)
Supplement: S1 Checklist — (DOCX) [file pgph.0000758.s001.docx]

Inclusivity in global research

PLOS’ policy on inclusivity in global research aims to improve transparency in the reporting of research performed outside of researchers’ own country or community and ensures that PLOS publications reporting global research adhere to high standards for research ethics and authorship. Authors of relevant research articles may be asked to complete the questionnaire below, which outlines ethical, cultural, and scientific considerations specific to inclusivity in global research. This questionnaire may be requested when researchers have travelled to a different country to conduct research, if research uses samples collected in another country, research with Indigenous populations or their lands, or if research is on cultural artefacts. Researchers travelling to another country solely to use laboratory equipment will not normally be required to complete the questionnaire. However, the questionnaire can be requested at the journal’s discretion for any submission – if you have been requested to complete this questionnaire by the PLOS journal you submitted to, please do so.

Please complete the questionnaire below and include this as a Supporting Information file with your manuscript. Note that if your paper is accepted for publication, this checklist will be published with your article in the supporting information files. Please ensure that you reference the checklist in the main body of your manuscript. We suggest adding a subsection ‘Inclusivity in global research’ to your Methods section and adding the following sentence: “Additional information regarding the ethical, cultural, and scientific considerations specific to inclusivity in global research is included in the Supporting Information (SX Checklist)”

The questions have been designed to be applicable to a wide range of study types, and there are subsections for both human subjects research and non-human subjects research. If any of the questions are not relevant to your research please mark them as “N/A” as appropriate.

**Ethical considerations, permits and authorship**

*This section is applicable to all research types.*

Provide details as to who granted permissions and/or consent for the study to take place in the Methods section of your manuscript. This should include the names of **all** ethics boards, governmental organizations, community leaders or other bodies that provided approval for the study. If individuals provided approval refer to these people by their role or title but do not list their name(s).

Reported on page number: 13 (Section 4.2 Ethical approval and consent to participate)

If there were any deviations from the study protocol after approval was obtained please provide details of these changes in the Methods section of your manuscript.
Did this study involve local collaborators that are residents of the country where the research was conducted or members of the community studied? If you do not have any authors from said communities, please provide an explanation for this below.

Reported on page number: not applicable

Yes. This forms part of a larger interdisciplinary research project ([MonkeyFeverRisk](https://www.monkeyfeverrisk.ceh.ac.uk/)) compising local collaborators from India who participated collaboratively in the project framing, research execution and writing of related mansucripts. Indian collaborators are contributing co-authors on the manuscript.

Everyone listed as an author should meet PLOS’ criteria for authorship and all individuals who meet these criteria should be included in the author byline, rather than the acknowledgements. Authorship criteria is based on the International Committee of Medical Journal Editors (ICMJE) Uniform Requirements for Manuscripts Submitted to Biomedical Journals - for further information please see here: <https://journals.plos.org/plosone/s/authorship>.

**Human subjects research (e.g. health research, medical research, cross-cultural psychology)**

Did you obtain written informed consent from a representative of the local community or region before the research took place? How did you establish who speaks for the community? Details of written informed consent obtained from study participants should be reported separately in the Methods section of your manuscript.

As part of our initial data collection plan, we engaged with the target local community groups and explained the overall purpose and objectives of our research project, their envisaged role and participation. The engagement was facilitated by colleague in India who have had lonstanding enaggeemnt with the focal community groups and have rick contextual knowledge of key community representatives. Based on the initial community engagement, we identified a list of potential participants for the household surveys and key infromant interviews who we subsequently invited to partyicipat in the study. As explained in Sections 4.2 and 4.3 of the manuscript, all participants gave their (verbal) consent to partipate in the study which wasduly recorded in field journals by the research team that administered the survey and interviews respectively. The rationale for obtainingverabl consent was principally due to the limited capacity of study particpatnts in the focal communities to read and write eithther in the local languages (Kannada and Malayalam) nor English.

How did members of the local community provide input on the aims of the research investigation, its methodology, and its anticipated outcome(s)?

As explained above, we adopted a co-production approach to our engagement with local stakehodlers to undertand their needs, priorities and co-develop solutions with respect to the research problem investigated. Through interactions during our initial community engagement phase and multi-stakeholder workshops, we collated feedback and inputs on the broader project aims, execution process and anticipated outputs. Based on the inputs and feedback collated, the project focus and stakeholder engagement plans were revised and operationalized. For interest, we have added a link to a recently published paper (<https://doi.org/10.1371/journal.pgph.0000075>) explaining in detail our co-production approach in the context of the MonkeyFeverRisk project.

When engaging with the local community, how did you ensure that the informed consent documents and other materials could be understood by local stakeholders?

Given the rich contextual knowledge and longstanding engagement of Indian colleagues with target local communities, we developed a community sensitive approach to our stakeholder engagement from nthe onset of the project. In this sense, all participant information sheets and consent forms were translated from English to the local languages (i.e. Kannada and Malayalam respectively) and vetted by research assistants conversant with the focal communities to ensure that the materials were comprehensible and captured the essence of the project focus. Prior to the actual survey and key informant interviews, we undertook a pilot survey which afforded the field research team the opportunity to trial our survey questionnaire/ interview guide and participant information sheets with a selected group of participants in the focal communities. Based on feedback from the pilot survey, we further refined the participant information sheet, survey questionnaire and interview guide.

In administering the surveys and key informant interviews, the field research team also read out and verbally explained the project aims and consent procedures to participants at the beginning and end of each interview/survey. The field research team also provided their telephone contact details to each participant in the event that they had follow up questions or clarifications about their participation and/ or the broader project. Where feasible, our field research team collaborated with local tribal promoters and community assisted professionals (who are embedded within the communities and well versed with the local customs and protocols) in further explaining the project consent processes to selected key informants. Details fof our consent process has been resported in Sections 4.2 and 4.3 of the manuscript.

Will the findings of the research be made available in an understandable format to stakeholders in the community where the study was conducted (e.g. via a presentation, summary report, copies of publications, etc.)? Please provide details of how this will be achieved.

Based on the co-production approach adopted for the broader MonkeyFeverRisk project, we have developed a variety of communication outputs including policy briefs, workshop resports, risk awareness leaflets, videos in Kannada and Malayalam, the predominant local languages in Shimoga and Wayanad respectively. These project communication materials have been made available through the Department of Health and Family Welfare (DHFWS) of Karnataka state and the Virus Diagnostic Laboratory Shimoga to local communities, disease managers and policy decision-makers at the local, district and state levels respectively. In particular, we developed tick information leaflets, risk guidance leaflets and videos for the local communities at risk of the Kyasanur Forest Disease based on the notion that findings of the research should be communicated in manner that is contextually appropriate and easy to understand. For interest, we have provided here a link (<https://www.monkeyfeverrisk.ceh.ac.uk/decision-support-tools-and-risk-guidance>) to our project webpage with additional information on the decision support tools, risk guidance and other communication materials from the project.

**Non-human subjects research using specimens/ animals collected as part of the study, or those housed in archival collections. Examples include archaeology, paleontology, botany and zoology.**

Did the permission you obtained from a local authority to perform the study include an agreement on access to outputs and benefit sharing? This may include procedures to enable fair distribution of the benefits and resources arising from the research performed. Please include any details of Prior Informed Consent and Benefit Sharing Agreements obtained. These may be required by field-specific regulations, for example the Convention on Biological Diversity (CBD) and the associated Nagoya Protocol.

Not applicable.

If the material used in your study was imported, please A) provide the year it was imported and B) indicate whether permits were obtained to import/export the materials used, C) provide details of any permits obtained. If this information is not available, please indicate this.

Not applicable.

If you used archival specimens, please state how the material used in your study was acquired by the institute it is held in and provide details of any permits obtained for the original excavations/ sample collection. If this information is not available, please indicate this.

Not applicable.

How was the potential cultural significance of the materials collected in your study to local communities considered in your research design? Were Indigenous peoples and/or local researchers and institutions involved with archaeological excavations / collection of specimens? If so, please provide a description of their involvement.

Not applicable.

If your manuscript includes photographs of human remains please indicate whether authors obtained permission from descendants or affiliated cultural communities to do so.

Not applicable.
