# Supplementary material for: The role of social vulnerability in improving interventions for neglected zoonotic diseases: The example of Kyasanur Forest Disease in India
Source: PLOS Glob Public Health. 2023 Feb 8;3(2):e0000758. doi: 10.1371/journal.pgph.0000758 (PMC10021172; doi:10.1371/journal.pgph.0000758)
Supplement: S1 Table — (DOCX) [file pgph.0000758.s002.docx]

**S1 Table.** Main thematic analysis results summaries based on key informant interviews with KFD survivors, district and taluka managers regarding their experiences and perceptions about 2018/19 KFD outbreak in the Western Ghats area of India.

| **Main Themes** | **Sub-themes** |
| --- | --- |
| 1. Knowledge and awareness of KFD | Misconceptions about KFD and imperfections in information delivery |
| 1. Human activities in ecosystems (vulnerability) | Complex trade-off between restricting forest access to minimise risk of exposure and safeguarding local livelihoods |
| 1. Prevention of tick-bites on people through of personal protective measures | Limited usage of DMP oil and uptake of other recommended personal protection measures |
| 1. Social and cultural barriers to uptake of current vaccine and personal protection measures | Pain and discomfort concerns with existing vaccine |
|  | Underlying religio-cultural sentiments and practices |
|  | Anxiety caused by lack of knowledge about KFD and its transmission pathways |
|  | Trust and legitimacy concerns |
| 1. Techno-administrative barriers to uptake of current vaccine and improvement considerations | Vaccination coverage and availability |
|  | Concerns about the efficacy of existing vaccine |
|  | Multiple vaccine does a disincentive. |
| 1. Impacts of KFD on affected individuals and households | KFD exposure amplifies other pre-existing health conditions |
|  | Changes in traditional livelihood organisation and practices - Reduction in number of days residing in forest and wearing of footwear in pursuit of livelihood options |
|  | Tribal groups often blamed as villains rather than victims |
|  | Eminent threat to existing livelihood organisation (fear and potential identity crisis as a maladaptation effects) |
