# Supplementary material for: The role of social vulnerability in improving interventions for neglected zoonotic diseases: The example of Kyasanur Forest Disease in India
Source: PLOS Glob Public Health. 2023 Feb 8;3(2):e0000758. doi: 10.1371/journal.pgph.0000758 (PMC10021172; doi:10.1371/journal.pgph.0000758)
Supplement: S2 Table — (DOCX) [file pgph.0000758.s003.docx]

S2 Table. Key informant interviews.

| **Department** | **Designation of participants** | **Number of participants (Shivamogga)** | **Number of participants**  **(Wayanad)** | **Level of operation**  (District /Taluk/ Local) |
| --- | --- | --- | --- | --- |
| Animal Husbandry | District officers | 1 | 1 | District |
|  | Animal health services manager | 1 | - | Taluk |
|  | Taluk official | 1 |  | Taluk |
| Health & Family Welfare | District health officials | 4 | 1 | District |
|  | Senior health worker | 1 |  | Taluk |
|  | Medical officer | 1 | - | Local |
|  | ASHA worker | - | 1 | Local |
| Local community | Tribal leaders |  | 2 | N/A |
|  | Farmers | 2 | 1 | N/A |
|  | Plantation workers | 3 | 1 | N/A |
|  | House wives | 3 |  | N/A |
|  | Forest-watchers | 1 | 1 | N/A |
| **Total** |  | **17** | **8** |  |
